# Microbial features of mature and abandoned soils in refractory clay deposits

**DOI:** 10.1186/s12866-022-02634-7

**Published:** 2022-10-04

**Authors:** Aleksei Zverev, Anastasiia Kimeklis, Arina Kichko, Grigory Gladkov, Evgeny Andronov, Evgeny Abakumov

**Affiliations:** 1 Saint-Petersburg State University, Saint-Petersburg, Russia; 2 All-Russia Research Institute for Agricultural Microbiology, Saint-Petersburg, Russia

**Keywords:** 16S amplicons, Abandoned soils, Anthropogenically disturbed soils, Microbiome analysis, Soil biodiversity, Soil microbiome

## Abstract

Processes of soil restoration in anthropogenically disturbed soils is an urgent topic in modern ecology and nature management. Being mediator between mineral soil composition and plant vegetation, soil microbial community is important factor of soil restoration processes. Analysis of main soil nutrition components followed by 16S amplicon sequencing are sufficient methods for primary analysis of novel locations. Here is the primary analysis in a novel location in Northwest Europe (Russia). Main nutrition parameters (pH, P, Na and NH_4_^+^) and 16S rDNA Illumina amplicons were explored in abandoned soils from sandy pit quarry (2 sites) and refractory clay mining dumps (4 sites).

Microbial communities of mature soils and dumps are variable and different in terms both nutritional and microbial components. pH, N and TOC are strong predictors for microbial composition. Dumps of refractory clays pQ_2 are non-developed soils, highly acidic and form specific microbial community. Differences between dumps and mature soils in both pre-quaternary and quaternary soils are connected with specific bacterial taxa. Those taxa are connected more with plant composition, not the soil properties themselves. The exact changes in microbial community are unique for different soils and areas.

**Graphical Abstract:**

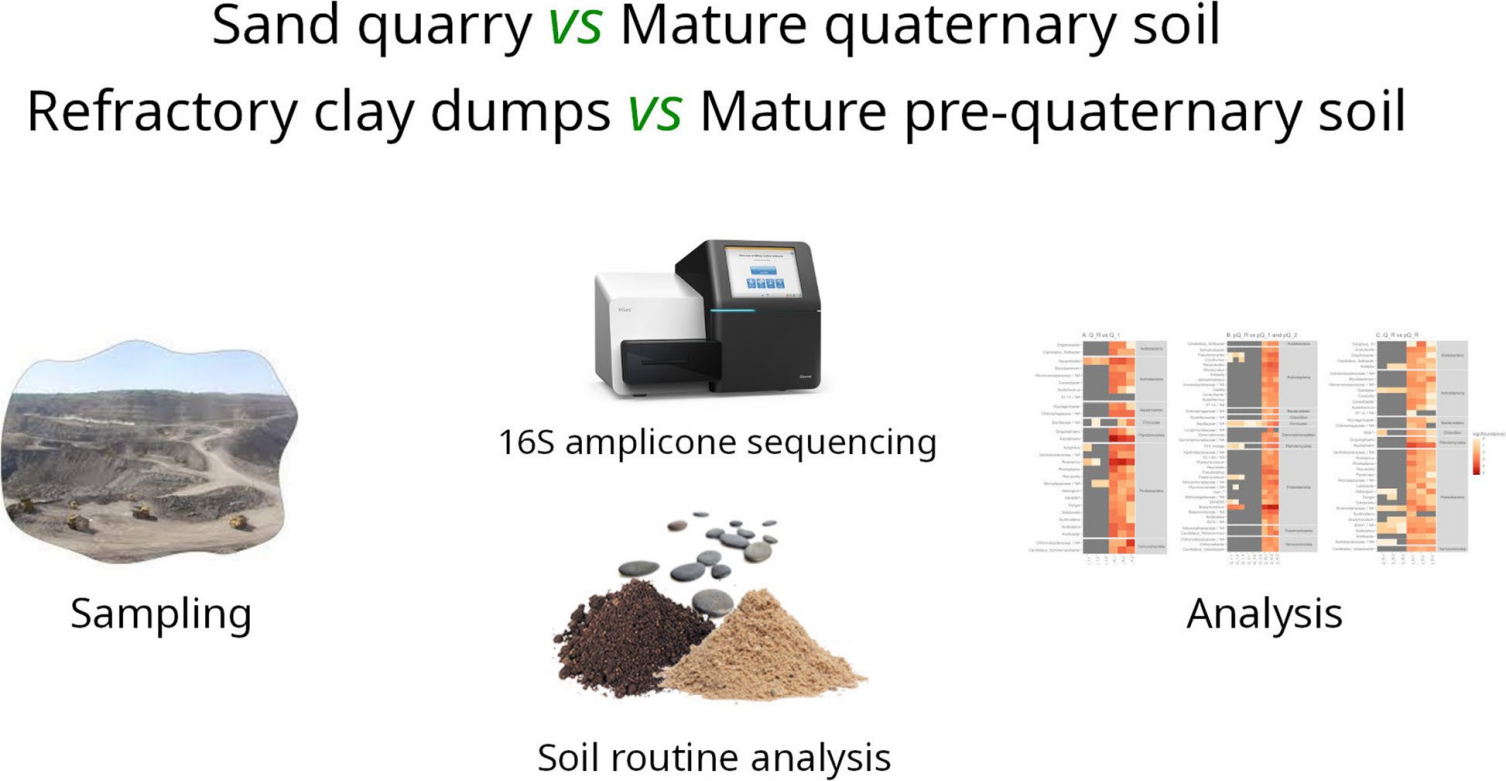

**Supplementary Information:**

The online version contains supplementary material available at 10.1186/s12866-022-02634-7.

## Introduction

Novgorod region is located in the west of the European part of the Russia. The diversity of minerals is especially great in the eastern part of the region, which contributes to intensive mining and disruption of the natural environment. Main substances mined in the region are refractory clays, sand and gravel.

Terrestrial ecosystems of industrial and urbanized territories are very different from natural landscapes. The urbanization process leads to a radical disruption of natural ecosystems, while the industrial development of resources, including mining, often ends with the complete destruction of the soil and vegetation cover [1, 2]. Partial transformation or significant disturbance of ecosystems is an urgent problem of modern ecology and nature management, which must be solved also by introducing of ecological management elements. Numerous challenges in environmental management are complicated by increasing of areas, occupied by open cut mines and heaps on the land surfaces [3, 4]. These heaps, exposed to effect of soil forming factors, could be considered as a model of primary soil formation [5]. Such a model serves as informative tool for evaluation of initial soil formation rate, biogenic-abiogenic interactions and restoration ecosystems [6]. At the same time, exposing on the surface of landscape of parent materials, which are unusual for current quaternary cover, result in developing of pedogenesis in complicated geogenic (lithogenic and topographic) conditions; namely, in overcomposed, hyperskeletic or toxic parent materials [7].

Exposure of mineral substrates of pre-quaternary genesis on the surface of quarry dumps and waste heaps leads to the alternative processing of initial soil, since in modern terrestrial ecosystems soil formation occurs mainly on rocks well processed by exogenous processes. Primary soils formed on non-regular rocks are very different from embryozems on quaternary substrates [8]. In this regard, it is interesting to study the morphological organization, chemical properties and microbiological parameters of primary soils and soil-like bodies formed on newly exposed dumps of various material and chemical composition. Morphological and chemical studies of the soils of various quarries were previously carried out [9] in various natural zones.

Microbial community is an important component in soil development processes. There are several studies of the microbiome of disturbed soils using high-throughput sequencing methods [5, 10, 11]. Despite it, this theme is far from generalization. Microbial shifts are unique for soils with different legacy and properties and should be investigated in multiple sources and processes. This research is dedicated to the exploration of microbial shifts in processes of soil restoration in a relatively novel location – abandoned refractory clays’ deposits in Northwest Europe.

## Materials and methods

Four sites were selected in the area of abandoned extraction of refractory clays (pre-quaternary soils pQ_1 – pQ_3 and pQ_R), and two sites in the area of abandoned extraction of sandy deposits (quaternary deposits Q_1 and Q_R): both locations were abandoned 20–30 years ago. The soils around of both the refractory clay deposit and the sand pit are represented by zonal variants (Albeluvisols, or retisols, according WRB 2015). In the first site the zonal soil is sod-podzolic light loamy soil on red-brown non-carbonate moraine sediments (Retisol Loamic). Refractory clay mining dumps are formed only by dumps of moraine material; no signs of horizon formation have been revealed on toxic soils or waste heaps (Primary soil or Entisols). The sandy quarry dumps belong to the embryonic soil (Entisol), characterized by a developed gray-humus horizon; however, signs of podsolization are not developed due to the short time of soil formation. Chemical parameters of soils and dump substrates are given in the Supplementary Table 1.

**Fig. 1:**
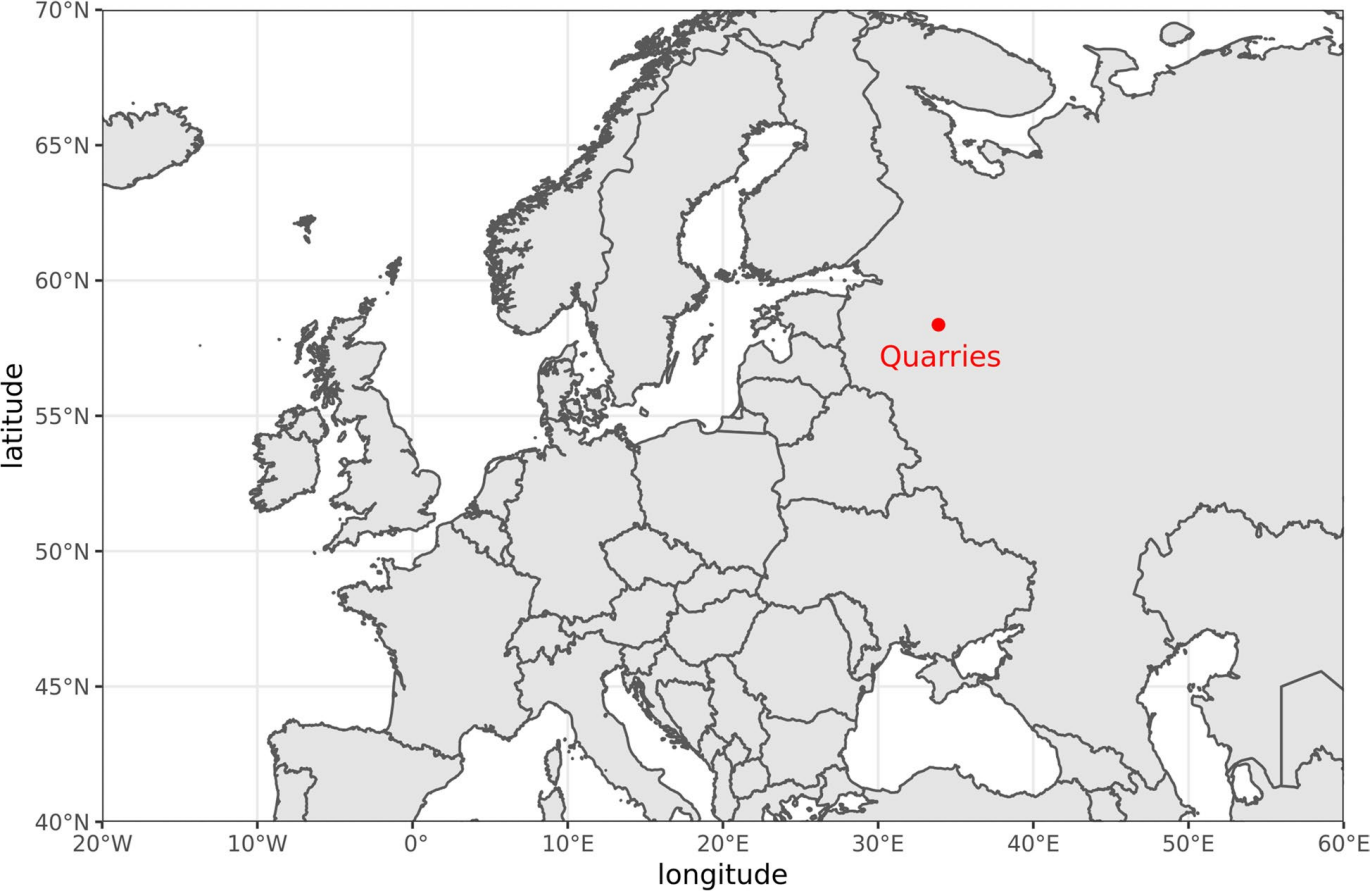
Global location of sampling areas

Samples were collected in 26^th^ of August, 2020 in 3 replicates from each spot. Description of sampling sites and their global location are in Table 1 and Fig. 1 respectively. Samples for microbiological analysis were collected from topsoil (in Table 1 the horizon of sampling is marked by asterisk), frozen, transported and stored at -20 °C. Samples for agrochemical analysis were collected in zip-lock plastic bags and stored at + 4 °C. For all samples main nutrition parameters were determined – pH [12], available phosphorus [13] and potassium, ammonium and nitrate nitrogen [14]. Total organic carbon (TOC) was determined using a CHN analyzer Leco CHN-628 (Leco Corporation, USA). Soil routine analyses were performed in accordance with Kimeklis et al., 2021 [5].

**Table 1:** Sample description

Name	Soil type	Local description	Plants	Horizons	Inclusions	Coordinates
**Refractory clay mining dumps (pre-quaternary sediments)**						
pQ_1 (Overburden Soil)	Loamy gray-humus on moraine dump overburden	Overgrown (about 20 years) heaps of quaternary overburden	Reed grass, clover, willow, peas, herd grass, yarrow (100% projective cover)	AY (0 – 7)*, C(7–18) Loosely riddled with roots	Loosely riddled with roots	58.366140, 33.877831
pQ_2 (Stone Heaps)		Lithostrat (heaped mineral ground), mix with large unrolled rocky debris	Absent	Dump overburden without features of pedogenesis formation	Roots, coal, oxidized pyrite	58.366330, 33.879089
pQ_3 (Spoil Heaps)		Waste heap (about 70 years old) at the coal mines	Absent, presumably due to extremely low pH	Dump overburden without signs of soil formation	Coal, pyrite	58.364520, 33.886705
pQ_R (Bulk Soil)	Sod-podzolic (Albeluvisol) developed on moraine heavy textured loams	Red-brown moraine outcrop, zonal south taiga meadow ecosystem	Scotch pine forest	AY (0 – 4)*, EI (4 – 10), BI (10 – 30)		58.365306, 33.888421
**Sandy quarry (quaternary deposits)**						
Q_1 (Sand Dumps)		Entisoil (embryonic soil) on sands (30 years), sand pit dumps	Young pine stand	AY (0 – 1)*, C (1 – 20)	a lot of litter	58.397174, 33.395037
Q_R (Bulk Soil)		Background podzol on glacial sands (over the road from the quarry)	Pine forest, spruce undergrowth, fern, oxalis	O (0 – 4)*, AY (4 – 7), E (7 – 10), C (10 – 20)		58.399094, 33.397613

* is marked the horizon of sampling

DNA was isolated using the MN FastDNA Spin Kit (MN, Germany) using a Precellus 24 homogenizer (Bertin, USA). The quality control of the isolation was carried out by PCR and agarose gel electrophoresis. Due well-developed methods of taxonomical annotation and relatively representative sequencing, the v4 variable region (f515/r806) of 16S rDNA gene was selected for future analysis. Sequencing of the variable region was performed on the Illumina MiSEQ sequencer using primers f515 (GTGCCAGCMGCCGCGGTAA) and r806 (GGACTACVSGGGTATCTAAT) [15]. The datasets generated and analyzed during the current study are available in the SRA repository, https://www.ncbi.nlm.nih.gov/sra/PRJNA705583

The general processing of sequences was carried out on the dada2 (v1.14.1) package [16]. Reads were filtered by length (240 bp for forwards and 180 for reverse) and expected errors’ rate (maxEE = 2) no N were allowed. Reads were paired by “consensus” method, and annotate using Bayesian Naive classifier using SILVA 134 database as the training set [17]. The main diversity analysis of the results was carried out using the phyloseq (v1.30.0) package [18] in R (v3.6.3). Differential abundance of taxa in pairwise comparisons was estimated using DESeq2 (v1.26.0) [19]. Difference in abundances (marking ASV as “variable”) was determined by two thresholds (baseMean >  = 10 and log2FoldChange >  = 2) and *p*-adj < 0.05. CCA analysis performed in vegan package (2.5–6) [20].

## Results

Nutritional properties of soils in common are typical for regional soils (Supplementary Table 1). Mature soils on moraine substrates are characterized by a neutral pH, while the background soil is slightly acidic. Spoil heaps are characterized by strongly acidic reaction (in this case, due to the presence of pyrite, oxidized to sulfuric acid and potassium sulfate). For both sandy soils acidic reaction of the environment is close to neutral. Low carbon and nitrogen content are typical for waste heaps and toxic rocks. In all explored samples most common form of nitrogen is ammonium. This indicates an active transformation of nitrogen-containing organic substances, which does not come into nitrification, which is reasonable for young soil formations. In substrates of quaternary origin there are more available phosphorus and potassium. (excluding toxic spoil heaps). Thus, the studied substrates are different in their initial physicochemical parameters and regimes of nutrients.

The total number of sequences after processing was 1 461 079 in 13 327 ASVs (Amplicon Sequence Variants). According results of pre-processing, the number of reads per sample for pQ_3 samples did not exceed 1500, and these samples were not used for further analysis. Alongside Bacteria, there were Archaea-attributed ASVs, but their abundance was less than 0.1%.

**Fig. 2:**
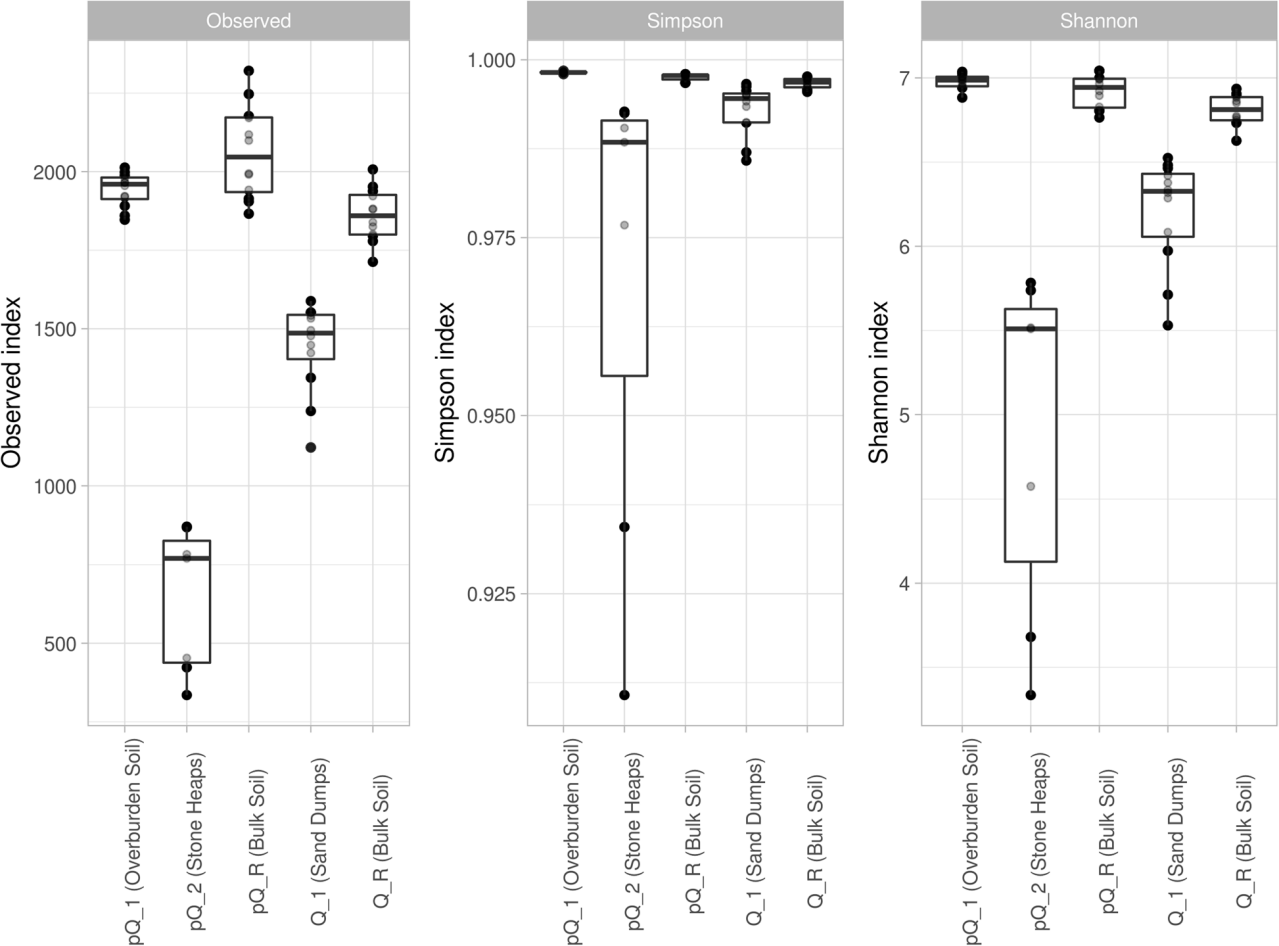
Alpha-diversity indices of microbial communities

Results of the analysis of alpha diversity are in Fig. 2. According to ANOVA, values of all indices are different in all groups (Observed: F = 85.6, *p* < 0.01, df_1_ = 4, df_2_ = 50; Simpson: F = 8.07, *p* < 0.01, df_1_ = 4, df_2_ = 50; Shannon: F = 45.39, *p* < 0.01, df_1_ = 4, df_2_ = 50). Alpha-diversity indices of samples pQ_1, pQ_R, Q_1, and Q_R is high (Fig. 2): the Simpson index approaches 1, the number of ASVs are not less than 1200. These samples are characterized by a relatively small range of indices’ values between replicates, which probably indicates general homogeneity of microbial community. Samples of group pQ_2, on the contrary, have a high range, as well as the small amount of ASVs.

**Fig. 3:**
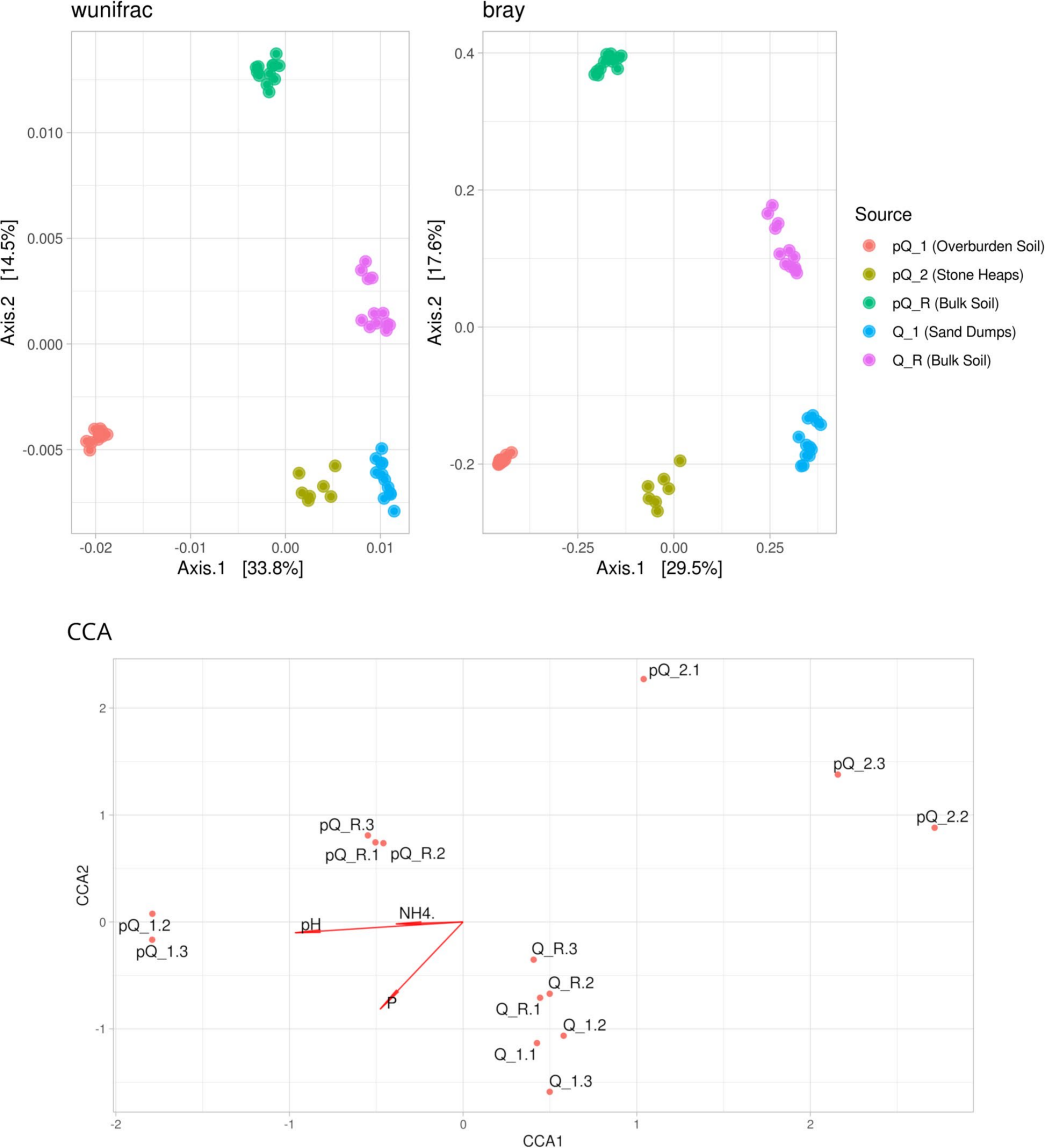
Beta-diversity indices of communities and CCA analysis (bray - PCoA of Bray distances; wunifrac - PCoA of Weighted UniFrac distances; CCA - CCA plot)

According to beta-diversity data (Fig. 3), all samples form clear clusters on the dendrogram, results of PERMANOVA shows sampling site as significant factor (bray: F = 24.7, *p* < 0.01, df_1_ = 4, df_2_ = 49; wunifrac: F = 23.3, *p* < 0.01, df_1_ = 4, df_2_ = 49). Agrochemical parameters fits well in sample distribution: according CCA analysis followed by permutational test, significant factors were pH, P and NH_4_^+^ amounts (Fig. 3).

**Fig. 4:**
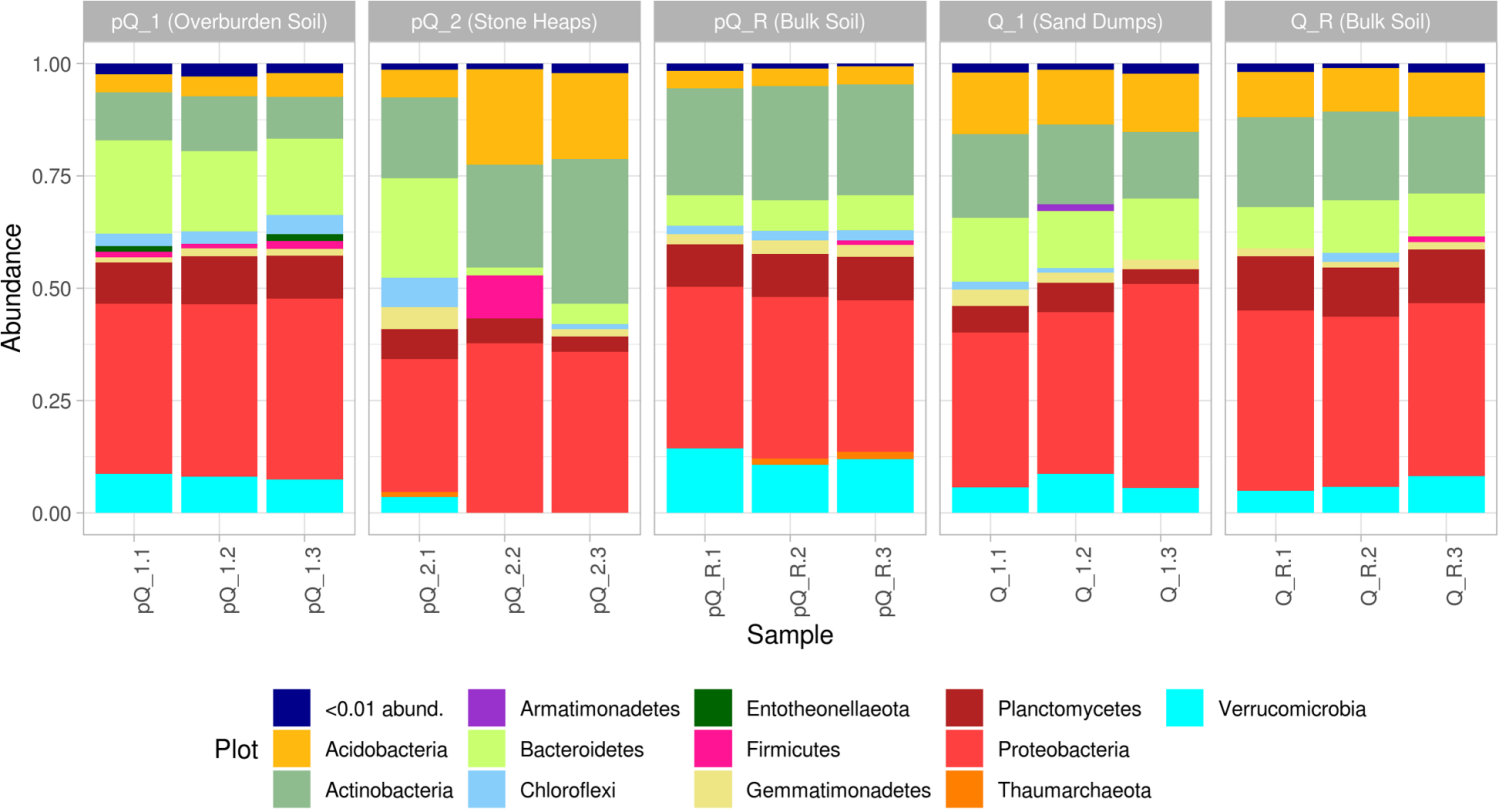
Relative abundance of bacterial taxa at phylum level (phyla, less abundant, than 0.01, are masked in “<0.01 abund.” group)

At phyla level, all samples demonstrate similar composition of taxa. The composition of pQ_R have more *Verrucomicrobia* and *Actinobacteria* species, and less *Acidobacteria* and *Bacteroidetes*. Q_1 samples demonstrate less *Planctomycetales*, than reference mature soil samples Q_R. Pre-quaternary samples pQ_1 have more *Bacteroidetes* and less *Actinobacteria*, than reference samples pQ_R, whereas pQ_2 samples are highly different by i) different taxonomical composition between replicas, ii) small amount of *Verrucomicrobia* and *Planctomycetes*, presence of *Firmicutes* (see Fig. 4).

**Fig. 5:**
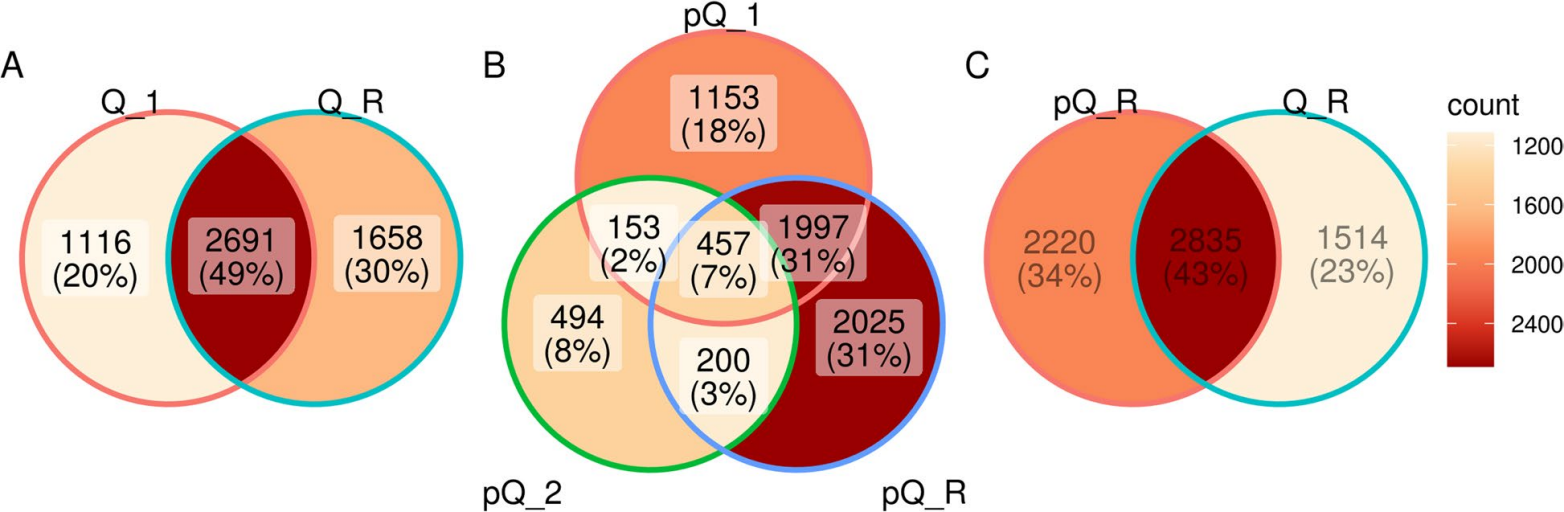
Common and unique shares of ASVs in comparisons of different soils

**Fig. 6:**
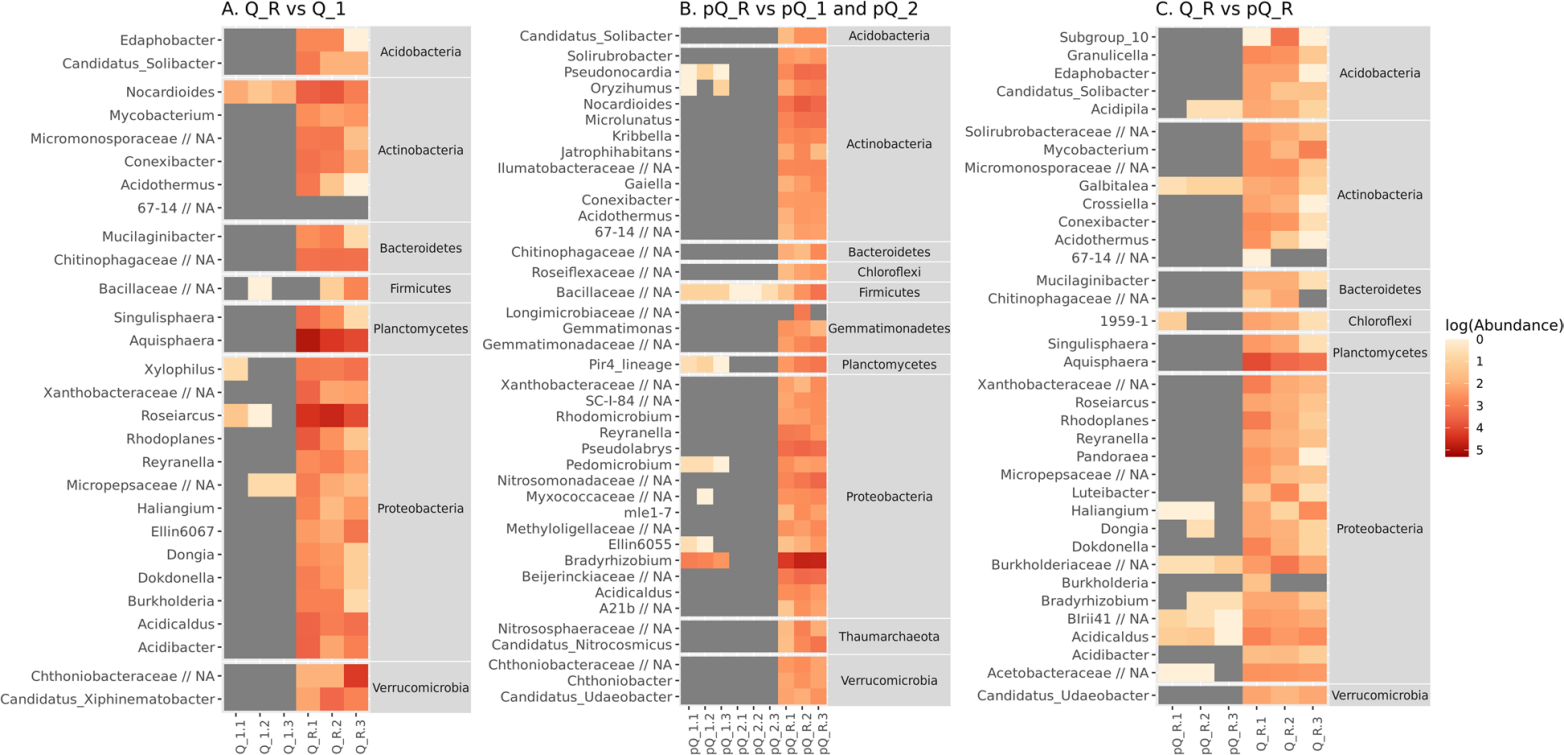
Significant differences in comparisons of the different soils

In comparison of unique and common ASVs in different soils the mature sandy (Q_R) and loamy (pQ_R) soils shares 43% of ASVs. 23% and 34% of ASVs are specific for both mature soils respectively. Sandy Q_1 soil have 20% ASVs as unique; pQ_1 and pQ_2 have demonstrate even less share of unique ASVs – 18% and 8% respectively (Fig. 5). More precise analyses at ASVs level reveal a range of taxa, which reflects significant differences between soil sites (Fig. 6).

## Discussion

Our results reveal significant differences between mature and dump soils. These differences can be explained primarily by early stage of succession, both plant and microbial. Another strong factor is the nutritional properties of substrate. Despite combination of multiple factors there is possible to investigate several aspects of soil formation.

*Well-developed soils are not similar in terms of microbial taxa, but functional similarity needs research.* The topsoil of both pre-quaternary sod-podzolic loamy soil (pQ_R) and podzol glacial sandy soil (Q_R) are active intermediates of well-established soil processes on same region. The main nutritional factors (except P amount) do not demonstrate significant difference. In terms of microbial composition both soils have diverse community without any difference in alpha-diversity (Fig. 1). It is reasonable to suggest, that microbial communities of both soils have i) relatively big amount of unique microbial taxa due different origin of microbial community, ii) predominance of similar or even same taxa due similar nutrition and development stage.

First thesis is supported by ASVs shares (Fig. 5), where up to 23% ASVs are unique for their soils. The second thesis is more complicated: relatively high distance in beta-diversity (Fig. 3) do not allows to find similarity in microbial communities. Despite it, the similarity possibly can be revealed in analysis of metabolic pathways. Another reason could be plant-soil interaction. Different plant communities form specific rhizosphere communities [21] which leads to differences in soil microbial community. Differential taxonomic analysis in Q_R and pQ_R comparison reveals mostly common plant-associated bacterial genera, like previously described *Aquispherae* [22], *Bradyrizobium* [23] and *Reyranella* [24]. In this case, the difference in soil communities are connected with plants, not the soil properties themselves. This theme needs additional research.

*Sand dumps are similar with mature soil, but in early stage of formation.* Embryonic sandy soils (Q_1) have similar origin with reference glacial sandy soil (Q_R), and have demonstrate similar nutrition condition (Supplementary Table 1). Relatively small Bray and Unifrac distances allows to suggest similar microbial community. Despite this similarity, sand dumps are undeveloped soil. Thin AY horizon, a lot of surface litter and monogenic pine overgrown are markers of early stage of soil formation. Amount of unique ASVs for this soil is 20% (and 116 ASVs), but according small beta-diversity distance these unique ASVs are minor components of microbial community. This suggestion is supported by differential expression analysis, where abundant ASVs were found mainly in Q_R reference podzol sands. These taxa also have been previously reported as the soil taxa connected with plants. The main differences were in abundance of *Aquisphaera* and *Roseiarcus*. *Aquisphaera* was previously reported in water microbiomes [22, 25] and forested tundra wetlands [26], *Roseiarcus* was described as part of blueberry rhizosphere microbiota [27]. In addition, *Edaphobacter* was previously isolated from alpine and forest soils [28], and *Xylophilus* was described in azalea plant in Korea [29]. *Acidibacter* and *Acidicaldus*, also revealed in this comparison, are both termo- and acidophilic ferric iron-reducing bacteria [30, 31], perhaps, are result of contamination.

*Dumps of refractory clays are toxic and form highly specific microbial community*. Overburden loamy soil pQ_1 have been derived from regular moraine as well as reference sod-podzolic loam pQ_R. Both of these soils have been overgrown by different plants up to 20 years and have clear soil horizons. As expected, alpha-diversity of microbial communities of both soils are high, soils share about 40% (2454 ASVs) of bacterial taxa. In the same time, beta-diversity distances between samples from both soils are extremely high. This pattern can be the consequence of different evolution of soils. Being formed on same basis, both soils have formed their own microbial communities with specific abundances of similar taxa.

In contrary, soil-like mix with large unrolled rocky debris (pQ_2) do not have any signs of soil formation or plant overgrow. Large rocks, coal and oxidized pyrite particles alongside highly acidic reaction (as a result of pyrite decomposition) leads to reducing of microbial activity. As a result, alpha-diversity from pQ_2 samples are low, both alpha-metrics and relative abundance at phyla level are variable, which indicates unstable bacterial community. Due 10% share of common microbial taxa, pQ_2 samples are highly specific. Despite this, differential abundance analysis does not found any acidophilic taxa, presumably due high variance in abundances. This research should be extended in a future by specific search of acidophilic taxa.

Due differential abundance analysis limitations, mainly common soil- and plant-associated taxa were found. Plant-associated microorganisms were founded previously in rhizospheres – for example, *Bradyrhyzobium* [23], *Nocardioides* [32]. Some of the taxa previously reported as typical soil microbiota – *Nocardioides* [32, 33], *Pseudonocardia* [34, 35], *Kribbella* [36], *Pseudolabrys* [37]. Except differential analysis limitation, this result can be connected with using of topsoil. The upper soil is closely connected with plants in well-developed soils, and do not have such connection in poor soils.

Due highly specific and complicated soil-microbial-plant interactions, the first stage of any research in a new location should be brief characterization of soil and microbial changes and setting of milestones for future research.

Determined nutritional factors—pH, carbon and nitrogen amount—are previously reported as the most important factors, shaping microbial community of restored soil [38–40]. Moreover, in some cases changes in soil pH are more important for microbial composition, than direct addition of nutrients [41].

Dumps and mine tailing soils in most researches are describing in different reclamation aspects. Time of soil exposure also can be viewed as a part of reclamation: according reports, reclamation time was the main force driving restoration of the soil quality and bacterial community [42]. In line with our results researchers reports, that soil bacterial and fungal community structures correlated mainly with vegetation density, and plant species [43]. The exact changes in microbial community are unique for different soils and areas. For example, in coal mining dumps relative abundance of *Proteobacteria*, *Actinobacteria* and *Bacteroidetes* was increased, while the one of *Acidobacteria*, *Chloroflexi* and *Nitrospirae* – decreased [42], while in our research the relative abundance of *Proteobacteria* were same, *Acidobacteria* and *Bacteroidetes* were ambivalent. The particular taxa included in restoration processes presumably also are unique for different soils: for example, in research of acidogenic mine tailings *Acidiferrobacter*, *Leptospirillum* (in location without plant overgrown) and *Bradyrhizobium* (in overgrown tailings) were reported as significant taxa. In our research *Bradyrhizobium* was found in mature soil. In acid fresh mine soils, the iron/sulfur-oxidizers such as *Acidiferrobacter* and *Sulfobacillus* were reported [44].

## Conclusions

Microbial communities of soils and dumps are variable and different in terms both nutritional and microbial components. PH, N and TOC are strong predictors for microbial composition.

Microbial communities of well-developed pre-quaternary and quaternary soils are different, possible functional similarity needs research. Dumps of refractory clays pQ_2 are non-developed, highly acidic and form specific microbial community. Differential abundance analysis does not found any acidophilic taxa, presumably due high variance in abundances. This research should be extended in a future by specific search of acidophilic taxa.

In comparison of dumps and mature soils in both pre-quaternary and quaternary soils we estimate specific bacterial taxa. They are connected more with plant composition, not the soil properties themselves. Except differential analysis limitation, this result can be connected with using of topsoil layer.

The exact changes in microbial community are unique for different soils and areas. Analysis of main nutrition components and 16S amplicon sequencing allow to find ways for more targeted research of different aspects and form specific hypothesis.

## Supplementary Information

**Additional file 1: Supplementary Table 1.** Agrochemical analysis of soils.

## Data Availability

The datasets generated and analyzed during the current study are available in the SRA repository, https://www.ncbi.nlm.nih.gov/sra/PRJNA705583.

## Abbreviations

ASV: Amplicon Sequence Variant
WRB: World Reference Base (for Soil Resources)
