# Supplementary material for: Microbial features of mature and abandoned soils in refractory clay deposits
Source: BMC Microbiol. 2022 Oct 4;22:237. doi: 10.1186/s12866-022-02634-7 (PMC9531468; doi:10.1186/s12866-022-02634-7)
Supplement: Supplementary file 1 — Additional file 1: Supplementary Table 1. Agrochemical analysis of soils. [file 12866_2022_2634_MOESM1_ESM.doc]

# Supplementary Table.1. Agrochemical analysis of soils.

|  | рН | P, mg/kg | K, mg/kg | N (from NH_4_^+^), mg/kg | N (from NO_3_^-^), mg/kg | TOС (total organic carbon %) | N (Total nitrogen %) |
| --- | --- | --- | --- | --- | --- | --- | --- |
| pQ_1.1 | 7.4 | 178 | 265 | 26.99 | <0.10 | 2.1 | 0.18 |
| pQ_1.2 | 7.5 | 185 | 252 | 28.33 | <0.10 |
| pQ_1.3 | 7.4 | 258 | 219 | 19.92 | <0.10 |
| pQ_2.1 | 4.3 | 16 | 223 | 3.23 | <0.10 | 0.32 | 0.02 |
| pQ_2.2 | 2.6 | 18 | 134 | 5.42 | <0.10 |
| pQ_2.3 | 3 | 12 | 92 | 8.29 | <0.10 |
| pQ_3.1 | 3 | 3 | 13 | 5.3 | <0.10 | 0.12 | 0.02 |
| pQ_3.2 | 2.8 | 6 | 21 | 4.39 | <0.10 |
| pQ_3.3 | 3 | 3 | 17 | 3.84 | <0.10 |
| pQ_R.1 | 5.2 | 80 | 370 | 46.54 | <0.10 | 2.38 | 0.15 |
| pQ_R.2 | 5.5 | 80 | 349 | 36.98 | <0.10 |
| pQ_R.3 | 5.4 | 52 | 332 | 27.29 | <0.10 |
| Q_1.1 | 5.2 | 170 | 126 | 16.14 | <0.10 | 0.45 | 0.06 |
| Q_1.2 | 4.8 | 166 | 122 | 0.18 | <0.10 |
| Q_1.3 | 5.1 | 229 | 126 | 13.16 | <0.10 |
| Q_R.1 | 4.3 | 104 | 303 | 57.87 | <0.10 | 0.25 | 0.14 |
| Q_R.2 | 4.4 | 120 | 429 | 24.31 | <0.10 |
| Q_R.3 | 4.3 | 83 | 235 | 19.74 | <0.10 |
